# Can a routine follow-up blood culture be justified in *Klebsiella pneumoniae* bacteremia? a retrospective case–control study

**DOI:** 10.1186/1471-2334-13-365

**Published:** 2013-08-02

**Authors:** Chang Kyung Kang, Eu Suk Kim, Kyoung-Ho Song, Hong Bin Kim, Taek Soo Kim, Nak-Hyun Kim, Chung-Jong Kim, Pyoeng Gyun Choe, Ji-Hwan Bang, Wan Beom Park, Kyoung Un Park, Sang Won Park, Nam-Joong Kim, Eui-Chong Kim, Myoung-don Oh

**Affiliations:** 1 Seoul National University College of Medicine, 103 Daehak-ro, Jongro-gu, Seoul, Republic of Korea 110-460; 2 Department of Internal Medicine, Seoul National University Bundang Hospital, 173-gil 82 Gumi-ro, Bundang-gu, Seongnam, Republic of Korea 463-707

**Keywords:** *Klebsiella pneumoniae*, Bacteremia, Risk factor, Follow-up, Blood culture

## Abstract

**Background:**

The need for mandatory confirmation of negative conversion in *Klebsiella pneumoniae* bacteremia (*Kp*B) has not been adequately addressed. We conducted a retrospective case–control study of adult patients with *Kp*B over a 5-year period in two tertiary-care hospitals to determine the risk factors for persistent bacteremia and to reevaluate the necessity of follow-up blood culture in *Kp*B.

**Methods:**

Persistent *Kp*B is defined as the finding of *K. pneumoniae* in more than two separate blood-culture samples for longer than a two-day period in a single episode. The case- and control-groups were patients with persistent and non-persistent *Kp*B, respectively, and they were matched 1-to-3 according to age and gender.

**Results:**

Among 1068 *Kp*B episodes analyzed after excluding polymicrobial infection and repeated *Kp*B, follow-up blood cultures were performed in 862 cases (80.7%), 62 of which (7.2%) were persistent. Independent risk factors for persistence were intra-abdominal infection, higher Charlson’s comorbidity weighted index score, prior solid organ transplantation, and unfavorable treatment response, which was defined as positivity for at least two parameters among fever, leukocytosis, and no decrease of C-reactive protein on the second day after initial culture. A proposed scoring system using four variables, namely, intra-abdominal infection, nosocomial *Kp*B, fever and lack of C-reactive protein decrease, the last two being assessed on the second day after the initial blood culture, showed that only 4.9% of the patients with no risk factors or with only intra-abdominal infection had persistent *Kp*B.

**Conclusions:**

Though persistent *Kp*B is uncommon, follow-up blood culture was performed in as many as 80% of the cases in this study. A more careful clinical assessment is warranted to reduce the cost and patient inconvenience involved in follow-up blood culture.

## Background

*Klebsiella pneumoniae* is one of the most important pathogens causing urinary tract infection, pneumonia, intra-abdominal infection, and primary bacteremia. It is also the second most common cause of community- and hospital-acquired gram-negative bacteremia [1-3]. Mortality from *Klebsiella pneumoniae* bacteremia (*Kp*B) is about 20 to 40% [3-5], and its population-based incidence ranges from 7.1 to 9.7 per 100,000 person-years [5,6].

There is evidence that the usefulness of routine blood cultures should be reconsidered in acute pyelonephritis [7,8], cellulitis [9], community-acquired pneumonia [10,11], and in isolated fever or leukocytosis [12]. Although attempts have been made to reduce unnecessary blood cultures by introducing clinical rules [12-14], clear guidelines have yet to emerge as to when blood cultures should be drawn. And blood cultures are liberally prescribed because of the high mortality due to bacteremia, anxiety about undertreatment, and fear of using inappropriate antimicrobial agents causing limited positivity of 4 to 7% [12,15,16].

Although it is widely appreciated that the requirement for follow-up blood cultures in bacteremic patients is questionable, they may still be prescribed too liberally, resulting in low rates of positive outcomes. Although routine follow-up blood culture is recommended in *Staphylococcus aureus* bacteremia and in infective endocarditis [17], the need for mandatory confirmation of negative conversion in *Kp*B has not been adequately addressed. The objective of the present study was to determine the risk factors for persistent bacteremia and to reevaluate the need for routine follow-up blood cultures in *Kp*B.

## Methods

### Study setting and patients

Data on all episodes of *Kp*B were collected from the electronic medical record systems between January 2007 and December 2011 at Seoul National University Hospital (a 1600-bed tertiary-care hospital, Seoul, Korea) and Seoul National University Bundang Hospital (a 900-bed tertiary-care hospital, Seongnam, Korea). All adult patients (≥ 18 years) who suffered their first *Kp*B episodes were enrolled. Cases of polymicrobial infection, and repeated episodes of *Kp*B in the same patient, were excluded.

Case- and control-patients were those with persistent *Kp*B and non-persistent *Kp*B, respectively. They were matched 1-to-3 according to age and gender using a greedy algorithm. The data reviewed included primary site of infection, Charlson’s co-morbidity weighted index [18], intensive care unit (ICU) admission during the episode of *Kp*B, severity of illness as measured by the Acute Physiology and Chronic Health Evaluation (APACHE) II score [19] and Pitt bacteremia score [20]; also microbiologic characteristics, both appropriateness of empirical antibiotic regimen and length of time from initial blood culture to antibiotic administration, as well as presence of fever, leukocytosis, and the trend of C-reactive protein (CRP) on the second day after initial blood culture. We also assessed the nosocomial or community onset of *Kp*B, presentation with septic shock, presence of metastatic infection, antibiotic or immunosuppressive therapy within the previous year, history of solid organ transplantation, any invasive procedure undertaken and neutropenia within 72 hours before the onset of *Kp*B, together with in-hospital mortality, and length of hospital stay.

The study was approved by the institutional review board of Seoul National University Hospital and Seoul National University Bundang Hospital.

### Definitions

The initial blood culture was defined as the first positive blood culture for *K. pneumoniae.* Persistent *Kp*B was defined as finding of *K. pneumoniae* in more than one separate blood cultures for longer than a 2-day period in a single infection episode. When an isolate was resistant to both cefotaxime and ceftazidime, it was suspected of being extended-spectrum *β*-lactamase (ESBL)-producing *K. pneumoniae*. A nosocomial infection was defined as an infection that occurred more than 48 hours after admission to hospital [21]. Fever and leukocytosis were defined as body temperature of ≥38°C and white blood cell count of >10,000 /μL. Neutropenia was defined as an absolute neutrophil count below 500/mL. Invasive procedures included insertion of a central venous catheter, endoscopy, endoscopic retrograde cholangiopancreatography, insertion of a nasogastric tube, bronchoscopy, parenteral nutrition, and insertion or manipulation of a percutaneous transhepatic biliary drainage [22].

Antimicrobial therapy was considered inappropriate if the isolate was not susceptible to the chosen antibiotic *in vitro*. The presence of at least two of the following: fever, leukocytosis, and lack of CRP decrease on the second day after initial blood culture, was pre-defined as an unfavorable treatment response before data collection.

### Microbiologic analyses

BacT/ALERT FA and FN (bioMe´rieux, Durham, North Carolina) was used for all blood cultures. Antimicrobial susceptibility was identified with a Microscan WalkAway-96 (Siemens Healthcare Diagnostics, Deerfield, Illinois) and VITEK 2 (bioMe´rieux, Marcy L’etoil, France), using the criteria of the Clinical and Laboratory Standards Institute (CLSI) guidelines. For suspected ESBL-producing *K. pneumoniae* isolates, ESBL production was confirmed by the double disk synergy test according to the CLSI performance standards [17].

### Statistical analyses

Conditional logistic regression was used in univariable and multivariable analyses to manage matched data. Variables with *P* values of < 0.10 in the univariable analysis or of clinical importance (i.e., intra-abdominal infection) were subjected to multivariable analysis. To evaluate the proposed scoring system, a receiver operating characteristic (ROC) analysis was carried out. *P* <0.05 was considered statistically significant. PASW for Windows (version 18 software package; SPSS Inc., Chicago, IL, USA) was used in the analyses.

## Results

### Rate of follow-up blood culture and incidence of persistent *KpB*

Of a total of 1372 *Kp*B episodes during the study period, 1068 were included in this study, after excluding 173 cases of polymicrobial infection and 131 cases of repeated *Kp*B. Among the 1068 enrolled cases of *Kp*B, follow-up blood cultures were performed in 862 cases (80.7%), and their incidence was highest on the second day after the initial blood culture (Additional file 1: Figure S1). Sixty-two (7.2%) were found to be persistent *Kp*B, and 186 non-persistent *Kp*B cases were matched with the latter as described above.

### Risk factors for persistent *KpB*

Univariable analysis of the data indicated that the factors significantly associated with persistent *Kp*B as opposed to non-persistent *Kp*B included higher Charlson’s comorbidity weighted index score, ICU admission, nosocomial infection, immunosuppressive therapy during the previous year, history of solid organ transplantation, invasive procedure during the previous 72 hours, fever and lack of CRP decrease on the second day after initial blood culture, ESBL-producing *Kp*B, inappropriate empirical antibiotic use, and in-hospital mortality (Table 1).

**Table 1:** **Univariable analysis for risk factors associated with persistent *Klebsiella pneumoniae *bacteremia (cases) vs. non-persistent *Klebsiella pneumoniae *bacteremia (controls)**

**Variables**	**Cases (n = 62)**	**Controls (n = 186)**	**OR (95% CI)**	***P***
Age (± SD)	63.77 (± 14.58)	63.58 (± 14.34)		
Male sex	45 (72.6)	135 (72.6)		
Urinary tract infection	7 (11.3)	20 (10.8)	1.05 (0.43-2.58)	0.908
Intra-abdominal infection	12 (19.4)	22 (11.8)	1.82 (0.83-4.00)	0.136
Biliary tract infection	13 (21.0)	53 (28.5)	0.65 (.032-1.32)	0.236
Liver abscess	8 (12.9)	31 (16.7)	0.73 (0.31-1.72)	0.469
Pneumonia	8 (12.9)	18 (9.7)	1.36 (0.57-3.20)	0.487
Primary bacteremia	9 (14.5)	34 (18.3)	0.73 (0.31-1.72)	0.474
Central venous catheter related infection	2 (3.2)	4 (2.2)	1.50 (0.28-8.19)	0.640
**Charlson’s CWI score (± SD)**	6.29 (± 3.05)	5.52 (± 2.94)	1.14 (1.01-1.28)	**0.037**
**ICU admission**	18 (29.0)	30 (16.1)	2.12 (1.08-4.16)	**0.029**
Presentation with septic shock	22 (35.5)	53 (28.5)	1.35 (0.75-2.44)	0.316
APACHE-II score (± SD)	18.97 (± 9.28)	17.09 (± 8.16)	1.03 (0.99-1.06)	0.140
Pitt bacteremia score (± SD)	2.45 (± 2.67)	2.17 (± 2.45)	1.04 (0.94-1.16)	0.452
**Nosocomial infection**	34 (54.8)	55 (29.6)	2.96 (1.61-5.43)	**<0.001**
Previous antibiotic use ≤ 1 year	37 (59.7)	95 (51.1)	1.42 (0.79-2.54)	0.240
**Immunosuppressive therapy ≤ 1 year**	5 (8.1)	4 (2.2)	4.46 (1.05-18.95)	**0.043**
**History of solid organ transplantation**	4 (6.5)	2 (1.1)	10.18 (1.11-93.02)	**0.040**
**Invasive procedure ≤ 72 hours before initial BC**	20 (32.3)	30 (16.1)	2.56 (1.29-5.07)	**0.007**
Neutropenia ≤ 72 hours before initial BC	11 (17.7)	31 (16.7)	1.08 (0.50-2.33)	0.843
Presence of metastatic infection	5 (8.1)	9 (4.8)	1.67 (0.56-4.97)	0.360
Inappropriate drainage	11 (17.7)	20 (10.8)	1.81 (0.80-4.07)	0.154
**Fever on the 2**^**nd **^**day after initial BC**	38 (61.3)	46 (24.7)	5.18 (2.64-10.16)	**<0.001**
Leukocytosis on the 2^nd^ day after initial BC	23 (37.1)	60 (32.3)	1.22 (0.68-2.19)	0.499
**Lack of CRP decrease on the 2**^**nd **^**day after initial BC**	26 (41.9)	43 (23.1)	2.38 (1.29-4.39)	**0.010**
**Unfavorable treatment response on the 2**^**nd **^**day after initial BC**	27 (43.5)	30 (16.1)	4.69 (2.27-9.69)	**<0.001**
**ESBL-producing *Klebsiella pneumoniae *bacteremia**	28 (45.2)	31 (16.7)	3.99 (2.09-7.63)	**<0.001**
**Inappropriate empirical antibiotic use**	22 (35.5)	24 (12.9)	4.02 (1.95-8.31)	**<0.001**
Length of time to empirical antibiotic administration from initial BC (minute, ± SD)	182.8 (± 221.3)	228.4 (± 434.8)	1.00 (0.99-1.00)	0.430
**In-hospital mortality**	19 (30.6)	34 (18.3)	1.96 (1.02-3.78)	**0.044**
Length of hospital stay (day, ± SD)	45.1 (± 43.0)	38.6 (± 74.9)	1.00 (0.99-1.01)	0.524

Data are no. (%) of patients, unless otherwise indicated.

OR, odds ratio; CI, confidence interval; SD, standard deviation; CWI, comorbidity weighted index; ICU, intensive care unit; APACHE, acute physiology and chronic health evaluation; BC, blood culture; CRP, C-reactive protein; ESBL, extended-spectrum *β* -lactamase.

Multivariable analysis revealed that the independent risk factors for persistent *Kp*B were intra-abdominal infection (adjusted odds ratio [aOR], 2.99; 95% confidence interval [CI], 1.07-8.31; *P* = 0.036), higher Charlson’s comorbidity weighted index score (aOR, 1.18 per each point; 95% CI, 1.01-1.36; *P* = 0.032), history of solid organ transplantation (aOR, 92.23; 95% CI, 1.27-6689.96; *P* = 0.038), and unfavorable treatment response (aOR, 4.79; 95% CI, 1.89-12.14; *P* = 0.001). The concept of unfavorable treatment response was introduced and substituted for fever, leukocytosis, and lack of CRP decrease in the multivariable analysis, to avoid the multicolinearity among those three variables. Neither ESBL-producing *Kp*B nor inappropriate empirical antibiotic use was found to be an independent risk factor (Table 2).

**Table 2:** **Independent risk factors for persistent *Klebsiella pneumoniae *bacteremia**

**Variables**	**Adjusted OR (95% CI)**	***P***
**Intra-abdominal infection**	2.99 (1.07-8.31)	**0.036**
**Charlson’s CWI score (± SD)**	1.18 (1.01-1.36)	**0.032**
ICU admission	1.98 (0.80-4.87)	0.139
Nosocomial infection	2.05 (0.81-5.21)	0.130
Immunosuppressive therapy ≤ 1 year	0.36 (0.02-8.90)	0.533
**History of solid organ transplantation**	92.23 (1.27-6689.96)	**0.038**
Invasive procedure ≤ 72 hours before initial BC	0.77 (0.27-2.19)	0.618
**Unfavorable treatment response on the 2**^**nd **^**day after initial BC**	4.79 (1.89-12.14)	**0.001**
ESBL-producing *Klebsiella pneumoniae* bacteremia	1.72 (0.63-4.69)	0.293
Inappropriate empirical antibiotic use	2.76 (0.93-8.23)	0.068

OR, odds ratio; CI, confidence interval; SD, standard deviation; CWI, comorbidity weighted index; ICU, intensive care unit; BC, blood culture; ESBL, extended-spectrum *β* -lactamase.

### Percentage of persistent *KpB* according to clinical score

To estimate the likelihood of persistent *Kp*B, a scoring system was introduced, based on the presence or absence of four risk factors viz. intra-abdominal infection, nosocomial *Kp*B, fever and lack of CRP decrease, the last two being assessed on the second day after initial blood culture. According to their odds ratio in a conditional logistic regression with these four risk factors, the weighted scores given for the risk factors were 1, 2, 3, and 2, respectively (Table 3).

**Table 3:** Odds ratios and adjusted weighted scores for the four variables used in the scoring system

**Variables**	**OR**	**Log (OR)**	**Adjusted weighted score**
Intra-abdominal infection	1.87	0.63	1
Nosocomial infection	3.24	1.18	2
Fever on the 2^nd^ day after initial blood culture	8.30	2.12	3
Lack of CRP decrease on the 2^nd^ day after initial blood culture	3.10	1.13	2

OR, odds ratio; CRP, C-reactive protein.

**Figure 1:**
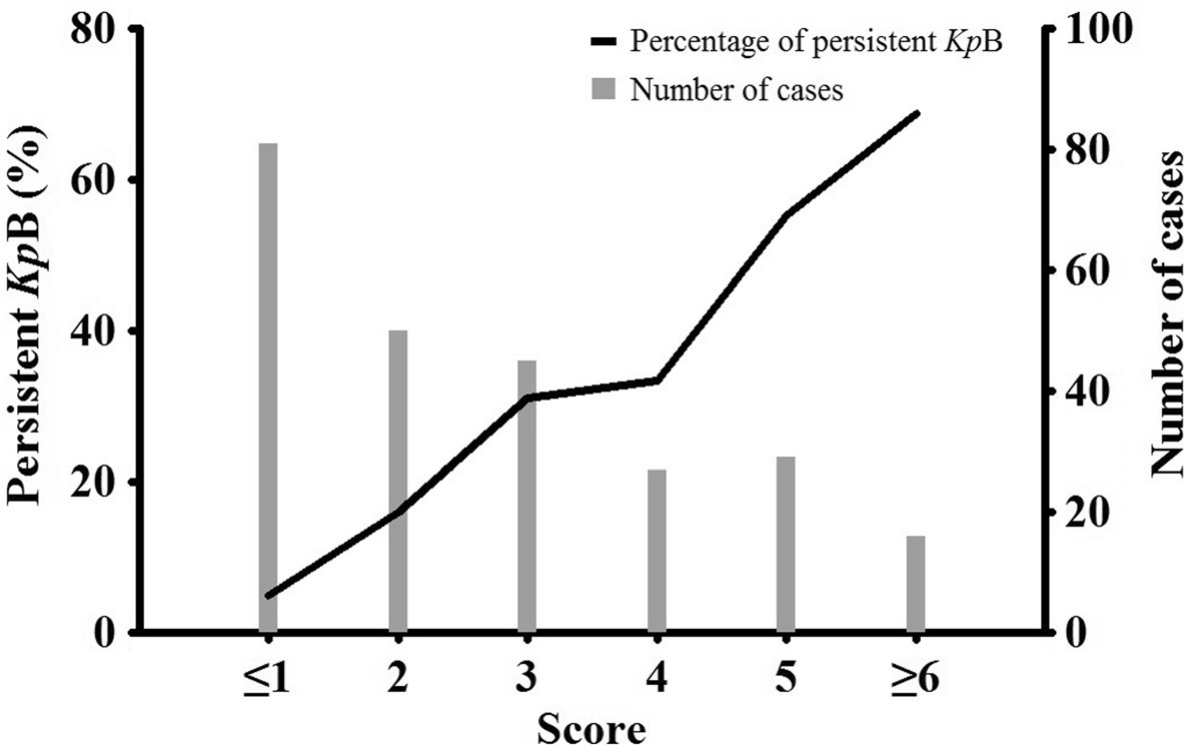
**Percentage of persistent *Klebsiella pneumoniae *bacteremia and number of cases according to clinical score.** There was a positive relationship between clinical score and percent persistent *Kp*B.

The frequency of persistent *Kp*B exceeded 50% when the score was greater than five; conversely, it was only 4.9% when the score was zero to one with no risk factors, or with intra-abdominal infection as the only risk factor (Figure 1). This indicates that persistent *Kp*B is likely to be rarer among patients with fewer risk factors. A ROC analysis showed that the area under the curve was 0.78 (*P* <0.0001) and the best cut-off was 2 (sensitivity, 0.81; specificity, 0.64, Additional file 2: Figure S2).

## Discussion

*K. pneumoniae* is one of the most important pathogens causing nosocomial and community-acquired infections, and clinicians sometimes encounter persistent *Kp*B in clinical practice. Despite the fact that the CLSI guidelines do not recommend routine follow-up blood cultures except in cases of *Staphylococcus aureus* bacteremia and infective endocarditis [17], our understanding of the occurrence of persistent *Kp*B and the frequency of follow-up blood cultures is far from complete. The present study showed that, although follow-up blood cultures were performed in 80.7% of the cases, only 7.2% were found to be persistent *Kp*B. Furthermore, more than two consecutive blood cultures were performed in 53.2% of the patients with non-persistent *Kp*B in the present study (Data not shown). These results imply that most of these follow-up blood cultures would have been unwarranted. Studies have shown that only 4 to 7% of blood cultures are positive owing to its liberal prescription [12,15,16], and our data with follow-up blood cultures also point to a similar situation. Our multivariable analysis revealed that unfavorable treatment response was an independent risk factor for persistent *Kp*B, along with intra-abdominal infection, high Charlson’s comorbidity weighted index score, and prior solid organ transplantation. All this suggests that, when treating suspected *Kp*B patients, the clinical findings should be considered carefully before opting for follow-up blood culture, to reduce indiscriminate prescription and the ensuing cost and patient inconvenience.

Severe underlying disease and prior organ transplantation are established risk factors for *Kp*B [4,5,23,24], as they were for persistent *Kp*B in this study. An intra-abdominal origin of *Kp*B is a known risk factor for mortality [4,5], and it was also significantly associated with persistent *Kp*B in our analysis. Although the *Kp*B of respiratory tract origin has been documented to be a risk factor for mortality [4,5,23], it was not significantly associated with persistent *Kp*B in this study. It would be interesting to find their association further by taking many factors into account, including the limitations of this study as explained below.

According to Kang *et al.*[25,26], ESBL-producing organism and inappropriate empirical antibiotic are not risk factors for increased mortality in bloodstream infections due to *K. pneumoniae.* We also found that production of ESBL was not an independent risk factor for persistent *Kp*B, which indicates that follow-up blood cultures are not routinely needed in cases of ESBL-producing *Kp*B. However, inappropriate empirical antibiotic regimen may call for further evaluation because of its borderline *P* value.

Various definitions of persistent bacteremia have been used in *Staphylococcus aureus* bacteremia [27-29]. Our definition of persistent bacteremia was not as strict as others because both the failure of appropriate antimicrobial therapy and prolonged periods of bacteremia were not essential elements of our consideration. If a stricter definition had been used, the incidence of persistent *Kp*B would have been further reduced, which supports our suggestion that more care should be taken before opting for follow-up blood culture.

Our scoring system, which has fair discriminative ability, could be used as a guide for whether a follow-up blood culture is needed, since there was a positive relationship between clinical score and percent persistent *Kp*B (Figure 1): the percentage of persistent *Kp*B declined sharply as the score decreased, and less than 5% of the patients with zero or one point had persistent bacteremia. On the other hand, follow-up blood culture could be justified in patients with more than five points because more than a half of those would be expected to suffer from persistent bacteremia. According to our data, if follow-up blood cultures were only carried out in patients with scores exceeding 2 points, more than 50% of follow-up blood cultures could be avoided (Figure 1).

There are some limitations to this study. There is a possibility of bias because it was a retrospective study with a limited sample size. Although our scoring system appeared to be useful, as discussed above, it should be further assessed on a larger sample. We were not able to evaluate the possible impact of age and gender on persistent *Kp*B by matching for these factors because of the design of this study. Also, there may have been selection bias in the 19.3% of patients without follow-up blood culture, one third of whom died by the 2nd day after blood culture (data not shown).

Persistent *Kp*B showed significantly worse outcomes, such as in-hospital mortality (*P* = 0.044) and ICU admission during the *Kp*B episode (*P* = 0.029) in univariable analyses (Table 1). However, we did not perform multivariable analysis for mortality-related factors, mainly because the study design was focused on persistence of bacteremia, and also the matching was carried out in that context.

Finally, this study was undertaken in university-affiliated tertiary-care hospitals where a large proportion of the patients might have severe underlying diseases including malignancy. However, 64.1% and 25.0% of our cases involved community-onset and community-acquired *Kp*B, respectively (data not shown). The rate of persistent *Kp*B would be expected to be much lower in a community-based hospital since a high Charlson’s comorbidity weighed index score is an independent risk factor for persistent *Kp*B and the comorbidities in patients in community-based hospitals are likely to be less severe.

## Conclusions

In conclusion, unfavorable treatment response on the second day after initial blood culture, intra-abdominal infection, high Charlson’s comorbidity weighted index score, and prior solid organ transplantation are independent risk factors for persistent *Kp*B. Since patients with persistent *Kp*B were rare, especially among individuals with few risk factors, routine follow-up blood culture may not be justified. More careful clinical assessment before deciding on follow-up blood culture would reduce costs and inconvenience to patients.

## Competing interests

The authors declare that they have no competing interests.

## Authors’ contributions

CKK, ESK, KHS, and HBK participated in the study design, data extraction, analysis of data, and writing of the manuscript. TSK, KUP, and ECK participated in data collection. NHK, CJK, PGC, JHB, WBP, SWP, NJK, and MDO advised on analysis and interpretation. All authors read and approved the final manuscript.

## Pre-publication history

The pre-publication history for this paper can be accessed here:

http://www.biomedcentral.com/1471-2334/13/365/prepub

## Supplementary Material

Additional file 1: Figure S1 Numbers of follow-up blood cultures, and numbers of those cultures positive for *Klebsiella pneumoniae* among the total of 1068 patients, according to days after the initial blood cultures. Click here for file

Additional file 2: Figure S2 Receiver operating characteristic analysis of the proposed clinical scoring system. Click here for file
