# Supplementary figures and images for: Can a routine follow-up blood culture be justified in Klebsiella pneumoniae bacteremia? a retrospective case–control study
Source: BMC Infect Dis. 2013 Aug 2;13:365. doi: 10.1186/1471-2334-13-365 (PMC3734211; doi:10.1186/1471-2334-13-365)

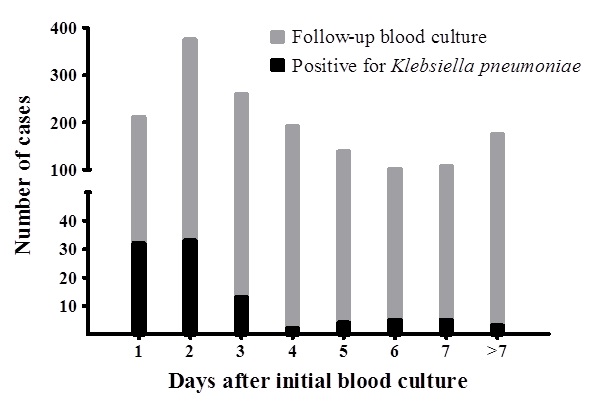

Supplement: Additional file 1: Figure S1 — Numbers of follow-up blood cultures, and numbers of those cultures positive for Klebsiella pneumoniae among the total of 1068 patients, according to days after the initial blood cultures. [file 1471-2334-13-365-S1.jpeg]

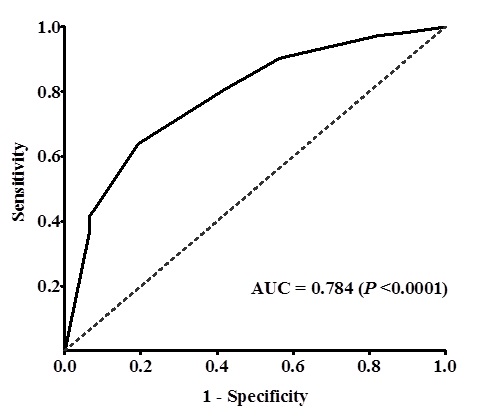

Supplement: Additional file 2: Figure S2 — Receiver operating characteristic analysis of the proposed clinical scoring system. [file 1471-2334-13-365-S2.jpeg]
